# Management of common mental disorders for psychogeriatric patients in Hong Kong: comparison of two clinics after 1 year of treatment

**DOI:** 10.1192/bjb.2018.13

**Published:** 2018-04

**Authors:** Mimi Mei Cheung Wong, Pui-fai Pang, Michael Gar Chung Yiu

**Affiliations:** United Christian Hospital, Hong Kong; email: wmc009@ha.org.hk

We would like to update the findings of our pilot study which compared the enhanced common mental disorder clinic (CMDC)^1^ and conventional specialist psychiatric out-patient clinic (SOPC) in the management of common mental disorders (CMDs) for psychogeriatric patients in our hospital in Hong Kong. In our previous letter to the editor, different clinical factors were compared between the two groups 6 months post-treatment. This time, findings for 1 year post-treatment were available.

The CMDC is a 1-year programme with multidisciplinary involvement. There were 30 patients in each group. After 1 year of treatment, only 15 patients (50%) remained in the CMDC, while 23 remained in the SOPC (*P* = 0.03). Ten patients (33.3%) completed the CMDC programme and were successfully discharged from the CMDC. They did not require any medication for their CMDs. One patient refused to attend medical follow-up. One patient was transferred from CMDC to SOPC, as she was found to have dementia. Another patient was transferred to the general out-patient clinic for continuation of treatment for her mixed anxiety and depressive disorder.

Concerning psychological intervention, half of the patients in the CMDC group (50%) were referred to a clinical psychologist, and ten had good adherence to appointments. For the SOPC group, only three patients (10%) were referred to a clinical psychologist (*P* < 0.05).

Concerning antidepressant use, the rate at 1-year follow-up was 11 (36.7%) for CMDC *v.* 20 (69%) for SOPC patients (*P* = 0.02). Profiles of benzodiazepine and hypnotic use and prescription were similar prior to consultation, after the first consultation, 6 months post-treatment and 1 year post-treatment. Eight patients (26.7%) in each group did not require benzodiazepines or hypnotics after the first consultation. The reduction in benzodiazepines and hypnotics was statistically significant (*P* = 0.04). At 1 year after treatment, ten patients (33.3%) in each group were taking benzodiazepines or hypnotics.

In summary, psychiatrists of both clinics were able to reduce benzodiazepine and hypnotic use after the patients formally presented to the psychiatric clinic. Nearly half of the patients who were given a benzodiazepine or hypnotic did not require it afterwards. Instead, about two-thirds of them were treated with antidepressants. Significantly more patients did not require antidepressants at 1 year post-treatment in the CMDC group. There were also significantly more patients who did not require medical follow-up at 1 year for the CMDC group.

Remission of CMDs is possible for patients who have completed the 1-year CMDC. Its psychological and pharmacological components, as well as timely interventions, have contributed to its success. The treating team and the patients are aware of the time-limited nature of the programme and have expectations that suitable cases can be discharged from the programme upon its completion. This helps to ensure that the clinic is not overwhelmed by continual accumulation of cases. On the contrary, the SOPC does not have a specified duration of treatment and the doctors are less ready to discharge patients from the clinic if they are stable on medical treatment. It is not common to refer back stable cases to primary care. Enhanced collaboration between the SOPC and primary care in the management of CMDs can help to reduce the burden on the SOPC, so that it can have more capacity to deal with complicated and unstable cases.^2^ The acceptability of psychological interventions is expected to be better if they can be more tailored to elderly patients; in fact, many older people expressed a preference for talking therapies.^3^
